# A Novel Mechanism of Transposon-Mediated Gene Activation

**DOI:** 10.1371/journal.pgen.1000689

**Published:** 2009-10-16

**Authors:** Zhongge Zhang, Milton H. Saier

**Affiliations:** Division of Biological Sciences, University of California San Diego, La Jolla, California, United States of America; Universidad de Sevilla, Spain

## Abstract

Transposable Insertion Sequences (IS elements) have been shown to provide various benefits to their hosts via gene activation or inactivation under stress conditions by appropriately inserting into specific chromosomal sites. Activation is usually due to derepression or introduction of a complete or partial promoter located within the element. Here we define a novel mechanism of gene activation by the transposon IS5 in *Escherichia coli*. The glycerol utilization operon, *glpFK*, that is silent in the absence of the cAMP-Crp complex, is activated by IS5 when inserted upstream of its promoter. High-level expression is nearly constitutive, only mildly dependent on glycerol, glucose, GlpR, and Crp, and allows growth at a rate similar to or more rapid than that of wild-type cells. Expression is from the *glpFK* promoter and dependent on (1) the DNA phase, (2) integration host factor (IHF), and (3) a short region at the 3′ end of IS5 harboring a permanent bend and an IHF binding site. The *lacZYA* operon is also subject to such activation in the absence of Crp. Thus, we have defined a novel mechanism of gene activation involving transposon insertion that may be generally applicable to many organisms.

## Author Summary

Transposons are “jumping genes” that can move from one location within a genome to another. Insertion of a transponson changes the DNA sequence and therefore gives rise to mutations that can activate or inactivate gene expression. Here, we demonstrate for the first time that one such transposon, Insertion Sequence 5 (IS5), when positioned upstream of a metabolic operon (*glpFK*) of *E. coli*, can activate the otherwise cryptic expression of the operon. This effect is due solely to a short region at the 3′ end of IS5 that harbors a permanent bend and an overlapping nucleoid protein binding site, both of which are required for maximal gene expression. We demonstrate the importance of phasing and conclude that DNA looping probably plays a role. We also show that another operon, the *E. coli* lactose operon (*lacZYA*), can be similarly activated by IS5. Although this is the first study to show that unique sequences within a transposon are necessary and sufficient to activate a downstream silent promoter, similar mechanisms of gene activation may occur for other operons.

## Introduction

Living organisms possess a variety of mutagenic means to generate genetic diversity, and these depend on environmental conditions and genomic composition [1],[2]. One frequently encountered type of mutation results from the insertion of transposable elements, transposons, which when inserted in appropriate locations of the genome, can activate or inactivate critical genes [3],[4]. One transposon-mediated mechanism of gene activation involves the formation of a “hybrid promoter” when a small transposon, an Insertion Sequence (IS) element, inserts into the promoter region. In this case, insertion of an IS element results in placing an outwardly directed −35 hexamer in one of the terminal inverted repeats (IRs) of the transposon at the correct distance from a resident −10 hexamer. Such −35 elements have been observed experimentally in several ISs [5]. Activation can also occur by initiating transcription within the transposon, traversing the terminal IR and reading the gene of interest. This second type of mechanism has been observed for IS3 [6] and IS10 [7].

A distinct type of gene regulation by ISs is illustrated by activation of the normally cryptic β-glucoside (*bgl*) catabolic operon in *E. coli*. Activation of this operon can be accomplished in several ways, one of which involves insertion of either IS5 or IS1 upstream or downstream of the promoter [8],[9]. For *bgl* operon activation, IS5 need not be in a specific position and orientation [9],[10], and the nucleoid structuring protein H-NS is required [11],[12].

The IS5 element has been found to activate the *fucAO* promoter [13], the *flhDC* promoter [14], and the *ade* promoter [15]. In wild type (*wt*) *E. coli* cells, expression of the *fucAO* operon is dependent on FucR, an activator of the *fuc* genes, and the cyclic AMP receptor protein (Crp). Prolonged incubation of *wt* cells with L-1,2-propanediol (which does not activate FucR) produced mutants that could grow on this carbon source. These mutants express *fucAO* independently of FucR and harbor IS5 inserted upstream of the *fucAO* promoter, always in the same orientation with its *ins5A* promoter distal to *fucA* [13]. In the case of *flhDC*, motility of *wt* cells on semisolid agar is substantially enhanced when IS5 is inserted at either of two locations (−99.5 and −169.5) upstream of the transcriptional start site. The IS5 orientations proved to be the same, with the *ins5A* promoter distal to the downstream *flhDC* promoter [14], as in the case of IS5 insertion in the *fucAO* promoter. In addition, IS5 has been shown to activate the cryptic *ade* gene encoding an adenine deaminase that catalyzes deamination of adenine to hypoxanthine in *E. coli* [15]. In the cases of *flhDC* and *ade*, activation is proposed to be due to the relief of HNS-mediated normal repression by IS5 insertion [14],[15] but the mechanism of IS5 activation of the *fucAO* promoter is unknown [13].

**Figure 1:**
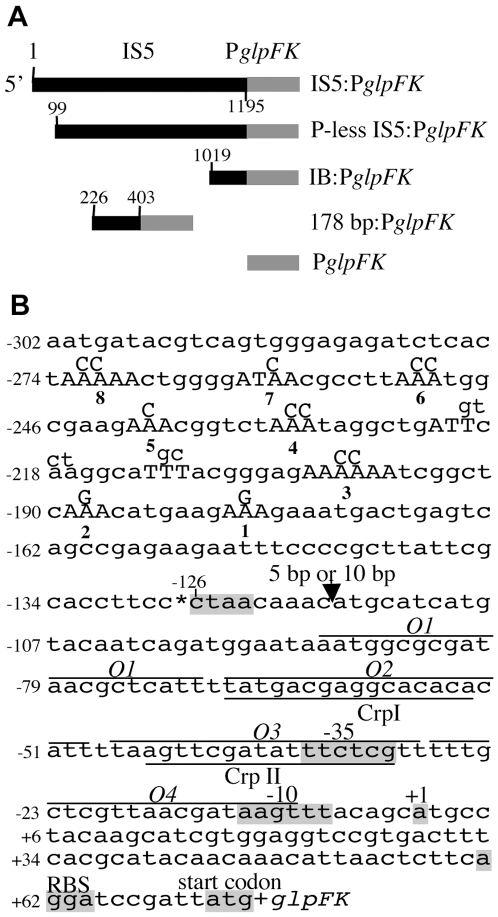
Schematic diagram of IS5 and its various regions upstream of the *glpFK* promoter (P*glpFK*) region used in this study (A), the 177-bp region (IB) at the 3′ end of IS5 and the *glpFK* promoter region (B). In (A), the numbers are relative to the 5′ end of IS5. IS5, P-less IS5, IB, and 178 bp refer to the entire IS5, IS5 deleted for the 98 bps from the 5′ end, the 177 bp 3′ end region of IS5 (IB), and the 178 bp region between the 226th and the 403rd nucleotides of IS5, respectively. In (B), the junction between IB and P*glpFK* is marked by an *. In IB, A-tracts are capitalized and numbered, and the IHF binding site is underlined. Mutations introduced into the IHF binding site or the A-tracts are labeled with letters above the sequence. In P*glpFK*, the +1, the promoter region (−10 and −35), the ribosome binding site (RBS) and the start codon for *glpF* are shaded. The GlpR binding sites are overlined and the Crp binding sites are underlined. The IS5 insertion site (CTAA) is shaded.

*E. coli* can use glycerol, glycerol-3-phosphate, or glycerophosphodiesters as sole carbon and energy sources. The loss of Crp abolishes its growth on glycerol. This is due to the fact that expression of one operon in the *glp* regulon, *glpFK*, encoding proteins essential for glycerol utilization [16],[17], is strongly dependent on the Crp-cAMP complex (Figure 1) [18],[19]. In addition, *glpFK* expression is repressed by the binding of GlpR, the *glp* regulon repressor, to the four operators in the *glpFK* promoter region (Figure 1) [20]. Repression is relieved in the presence of glycerol-3-phosphate, the inducer of the *glp* regulon.

Our previous research [21] demonstrated that *crp* deletion mutants can mutate specifically to utilize glycerol (Glp^+^). The frequencies of such mutations are enhanced by the presence of glycerol and decreased by GlpR. Of the four GlpR operators (*O1*-*O4*) upstream of *glpFK*, *O1* primarily controls mutation rate while *O4* specifically controls *glpFK* expression. All Glp^+^ mutants contain an IS5 upstream of the *glpFK* promoter, always in the same position and orientation [21].

Here, we describe a novel mechanism of gene activation due to IS5 insertion upstream of the promoter of the *glpFK* operon. Activation is so strong that it overcomes the repression resulting from the absence of Crp. A 177-bp fragment at the 3′ end of IS5, proximal to the downstream promoter, is both necessary and sufficient for full activation. Although for activation, IS5 always inserts into the same position upstream of the *glpFK* promoter, the activating effect is still observed when a 10 bp fragment, but not a 5 bp fragment, is inserted between IS5 and the *glpFK* promoter. Thus, the active element, encompassing a permanent bend and an IHF binding site, must be present at the correct phase angle relative to the promoter to be effective. Finally, it is shown that in the absence of Crp, the *E. coli* lactose operon can also be activated by upstream IS5 insertion. These results reveal a novel mechanism of gene activation by insertion sequences.

## Results

### Effects of IS5 Insertion on Expression of *glp* Genes in Various Genetic Backgrounds

**Figure 2:**
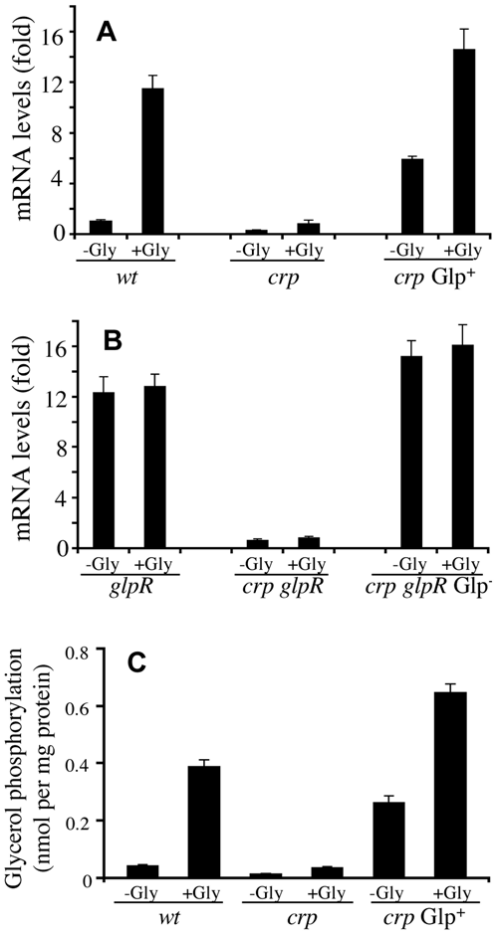
Real time PCR analysis of *glpFK* expression (A,B) and determination of glycerol kinase (GlpK) activity (C). (A) *glpFK* mRNA levels in *wt*, *crp* and *crp* Glp^+^ cells. (B) *glpFK* mRNA levels in *glpR*, *crp glpR* and *crp glpR* Glp^+^ cells. (C) Glycerol kinase (GlpK) activities in *wt*, *crp*, and *crp* Glp^+^ cells. Cells were grown in LB with or without 1% glycerol (Gly). Radioactivity was measured by scintillation counting with 10 ml of Bio-safe II fluid.

Using real-time PCR, we determined mRNA levels of the five *glp* operons comparing *crp* Glp^+^ cells (with an IS5 insertion upstream of the *glpFK* promoter) [21] with parental *crp* cells. No differences were observed in expression levels of four of the five operons, but the *glpFK* operon showed a dramatic difference. Figure 2A shows expression of *glpFK* in *wt*, *crp* and *crp* Glp^+^ cells. In the absence of glycerol, *glpFK* expression was >50 fold higher in *crp* Glp^+^ cells than in *crp* cells (see columns 3 and 5 from the left side), showing that the IS5 insertion led to high level expression of this operon. In the presence of glycerol, *glpFK* expression increased in all cell types examined, but expression was the highest in *crp* Glp^+^ cells, about 15-fold higher than in the *crp* cells incubated under the same conditions. However, the degree of induction by glycerol in *crp* Glp^+^ cells was greatly decreased, suggesting that in these cells, repression by GlpR is weak (∼2.5 fold, see columns 5 and 6) compared to that in *wt* cells (>10 fold, see columns 1 and 2).

When the *glpR* gene was deleted from each of these three backgrounds, glycerol induction of *glpFK* expression was abolished (Figure 2B), indicating that increased *glpFK* expression in *wt*, *crp* and *crp* Glp^+^ cells in response to glycerol is solely due to the release of GlpR from the control region of the operon.

We measured *in vitro* glycerol kinase activity in *wt*, *crp* and *crp* Glp^+^ cells using [^14^C]glycerol as substrate. The highest levels of glycerol phosphorylation activity were observed in extracts of *crp* Glp^+^ cells cultured in LB medium either with or without glycerol (Figure 2C). Glycerol induction was observed in all three genetic backgrounds, with induction levels similar to those of the mRNA revealed by real-time PCR (see Figure 2A).

To test if IS5 activation of the *glpFK* promoter (P*glpFK*) is due to relief of repression by H-NS as observed for the *bgl* operon [11],[12] and the *ade* operon [15], we mutated the *hns* gene in *wt*, *crp* and *crp* Glp^+^ backgrounds. Using real-time PCR, we found that the *hns* mutation had only a slight (<0.2 fold) effect on *glpFK* expression in all three genetic backgrounds compared to the same cells without the *hns* mutation (Figure S1), indicating that IS5 activation of P*glpFK* in the absence of Crp is not due to relief of H-NS mediated repression.

### Determination of the Start Site of *glpFK* Transcription in *crp* Glp^+^ Mutants

**Figure 3:**
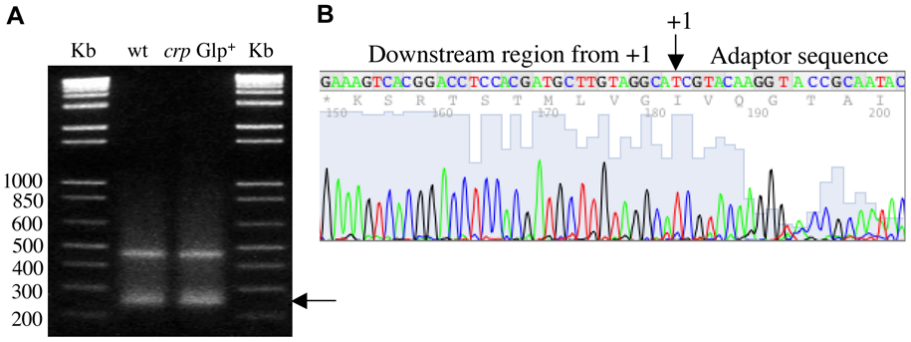
Analysis of the 5′ end of the *glpFK* message. (A) Gel electrophoresis of PCR products of the 5′ end of the *glpFK* cDNA. RNA ligase-mediated RT–PCR was employed to amplify the 5′ end of the *glpFK* mRNA. cDNA was synthesized using an Invitrogen superscript first-strand synthesis kit. The arrow points to the product resulting from a newly initiated message. The other band is a nonspecific PCR product. (B) Chromatogram of a part of the DNA sequence showing the junction between the 5′ end of the *glpFK* cDNA and the reverse transcribed adaptor. The amplified 5′ end of the *glpFK* cDNA was sequenced using the oligo PglpFK-extn-R (see Table S2) that binds to the ∼210 bp region downstream of +1. The arrow points to the transcriptional start site (+1) on the complementary strand.

To determine if the presence of the IS5 element provides a new promoter driving *glpFK* transcription, we measured the transcriptional initiation site of the operon using RNA ligase mediated RT-PCR. The total RNA was treated with tobacco acid pyrophosphatase (TAP) prior to cDNA synthesis. The 5′-end region of the *glpFK* cDNA was amplified using a pair of primers (Table S2), one (PglpFK-extn-F) binding to the adaptor sequence and the other (PglpFK-extn-R) binding to a region between 115^th^ and 137^th^ nucleotides downstream of the *glpF* start codon. Two PCR products were obtained for both the wild type and the *crp* Glp^+^ strain (Figure 3A). Using PglpFK-extn-R as primer, DNA sequencing showed that the larger product was non-specific; the smaller one was the 5′-end region of the *glpFK* cDNA product (Figure 3B). The junction was found to correspond precisely to the 5′ end mapped by primer extension (see Figure 1B) [18]. When the total RNA was not treated with TAP, only the nonspecific PCR product was obtained (see Figure 3A) for both the wild type and the *crp* Glp^+^ strains. Activation of transcription by IS5 in *crp* Glp^+^ cells is therefore driven by the native *glpFK* promoter. The start site was the same with or without glycerol (1%) in the growth medium.

### Effects of Different Regions within IS5 on *glpFK* Promoter Activity

**Figure 4:**
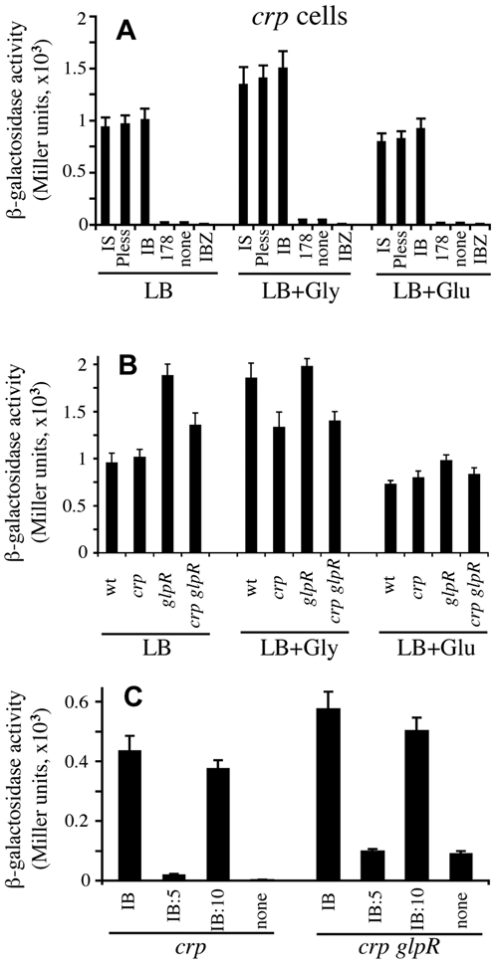
Control of *glpFK* operon promoter activity. (A) Effects of IS5, promoter-less IS5 and IB on expression of the downstream *glpFK* promoter in *crp* cells. ‘IS’, ‘P-less’, ‘IB’, ‘178’, ‘none’, and ‘IBZ’ refer to transcriptional *lacZ* fusions for IS5:P*glpFK*, promoter-less IS5:P*glpFK*, IB:P*glpFK*, 178 bp:P*glpFK*, native P*glpFK*, and IB alone, respectively (see Figure 1A). (B) IB:P*glpFK* activity in *wt*, *crp*, *glpR* and *crp glpR* cells. In both A and B, wild-type and mutant cells were grown with shaking in LB with or without 1% glycerol (Gly) or 1% glucose (Glu). (C) Effect of a 5- or 10-oligonucleotide insertion between IB and P*glpFK* on promoter activity. A 5 bp (TACCT) or a 10 bp (TACCTTACCT) fragment was inserted between −117 and −118 relative to +1 of P*glpFK* (see Figure 1B). β-Galactosidase activities of these promoters were measured in *crp* and *crp glpR* cells grown in minimal M9 medium + 0.66% casamino acids (CAA) + 1% glucose. ‘IB’, ‘IB:5’, ‘IB:10’, and ‘none’ refer to IB:P*glpFK*, IB:5 bp:P*glpFK*, IB:10 bp:P*glpFK* and P*glpFK* alone, respectively.

Using chromosomal *lacZ* fusions, we examined the effect of IS5 and different regions within IS5 on P*glpFK* activity (Figure 1A and Figure 4A). Consistent with the RT-PCR results, the presence of IS5 upstream of P*glpFK* (IS5:P*glpFK*) in *crp* cells dramatically increased the activity of the promoter regardless of the medium used. Both promoter-less IS5 (P-less IS5, in which the 98 bp 5′ end region containing the promoter of the transposase gene was deleted), and the 177 bp 3′ end region (called “Internal Bend”, IB) of IS5 activated P*glpFK* in *crp* cells to an extent comparable to that observed with the intact IS5. However, the 130 bp 3′ end region of IS5 (i.e., IB with 47 nucleotides removed from the 5′ end) activated the promoter about 40% less efficiently than the 177 bp IB region (data not shown, see below for explanation).

Addition of glycerol to LB medium further increased the promoter activity, in agreement with the conclusion that GlpR weakly decreased IS5:P*glpFK* activity. The increased level (∼1.5 fold) of P*glpFK* activity caused by the presence of glycerol in a *crp* genetic background, measured by β-galactosidase activity in a *glpF*-*lacZ* fusion strain, was lower than that observed (∼2.5 fold) for *glpFK* mRNA in a *crp* Glp^+^ background (Figure 2A). Such a difference may be due to the fact that *crp* Glp^+^ cells transport and subsequently phosphorylate glycerol more rapidly than *crp* cells. Addition of glucose to the medium slightly reduced the activities of the promoters tested, showing that the strong catabolite repression observed in the wild type strain was largely abolished. Further experiments reported below confirmed and extended this conclusion.

IB:P*glpFK-lacZ* activity was subsequently characterized in other genetic backgrounds. High levels of β-galactosidase activity were observed for *wt*, *crp*, *glpR* and *crp glpR* genetic backgrounds with cells grown in LB ± glycerol or ± glucose (Figure 4B). Similar levels of promoter activity were observed in *wt* and *crp* cells in the absence of glycerol, suggesting that the Crp protein does not appreciably influence IB:P*glpFK* activity. Addition of glycerol increased the overall IB:P*glpFK* activity by ∼2 fold in *wt* cells (column 1 and column 5 in Figure 4B) and ∼1.3 fold in *crp* cells (columns 2 and 6 in Figure 4B), while addition of glucose slightly decreased it. Higher promoter activities were detected in *glpR* and *crp glpR* cells than in *wt* and *crp* cells, respectively, in the absence of glycerol. Addition of glycerol did not further increase these activities in cells containing the *glpR* mutation (columns 4 and 8). However, in the absence of GlpR, deletion of the *crp* gene reduced the activating effect of IB by about 20% (Figure 4B). It is possible that Crp positively influences the expression of a gene encoding a protein such as IHF, which is required for full activation of P*glpFK* by IB. The role of Crp in IB activation of P*glpFK* thus appears to be minimal, but it could be indirect and complex.

As a control, a 178 bp internal fragment of IS5 (nucleotides 226 to 403 from its 5′ end), similar in length to that of IB (177 bps), was substituted for IB in IB:P*glpFK*, and the activity of the construct (178bp:P*glpFK*) was determined in *crp* cells. β-Galactosidase activity was not increased compared to P*glpFK* alone (Figure 4A). Another control showed that IB alone in front of *lacZ* (IB-*lacZ*) had no promoter activity regardless of the medium used (Figure 4A). The results presented in Figure 4A and 4B together show that the stimulation of P*glpFK* activity mediated by IS5 insertion is due to the IB segment (i.e., the 177 bases located at the 3′-end of IS5). Such activation is so strong that (1) Crp is not required and (2) GlpR only weakly represses.

To further show that the transposase, encoded by *ins5A* (981 bp) [22],[23], is not involved in IS5 activation, IS5:P*glpFK-lacZ*, promoter-less IS5:P*glpFK-lacZ*, IB:P*glpFK-lacZ*, P*glpFK*-*lacZ* and 178 bp:P*glpFK*-*lacZ* fusions were individually moved to a *crp* mutant background of *E. coli* strain B which lacks IS5 [24]. Similar to BW25113 *crp* cells, IS5 and IB equally elevated promoter activity in strain B *crp* cells compared to P*glpFK* alone (Figure S2). The 178-bp IS5 internal fragment was unable to activate P*glpFK* in these IS5-free *crp* cells. This experiment clearly demonstrates that the transposase does not contribute to IS5 activation of P*glpFK*. It is IB, the 3′ end region of IS5 that activates P*glpFK* in the absence of Crp.

### Effect of IB Positioning on the Activity of IB:P*glpFK*

To determine if IB activation of P*glpFK* is DNA phase dependent, two oligonucleotide sequences (5 bp and 10 bp) were individually inserted at −117.5 (relative to +1 of P*glpFK*) between IB and P*glpFK* (Figure 1). As shown in Figure 4C, insertion of a 5 bp nucleotide sequence almost completely abolished promoter activity in both *crp* and *crp glpR* cells although insertion of a 10 bp oligonucleotide sequence only slightly reduced promoter activity. These results indicate that the proper positioning of the upstream IB relative to P*glpFK* is essential for full promoter activation in *crp* cells. They are consistent with a requirement for a 10 bp periodicity in the position of the insert for strong stimulation as expected for B-DNA. The latter implies the involvement of DNA structure (e.g., bending or looping) in P*glpFK* activation by IB. These results were obtained for both IB and IS5 (data not shown).

### Requirement of IHF and the IHF Binding Site in IB for Full Activation of P*glpFK*

**Figure 5:**
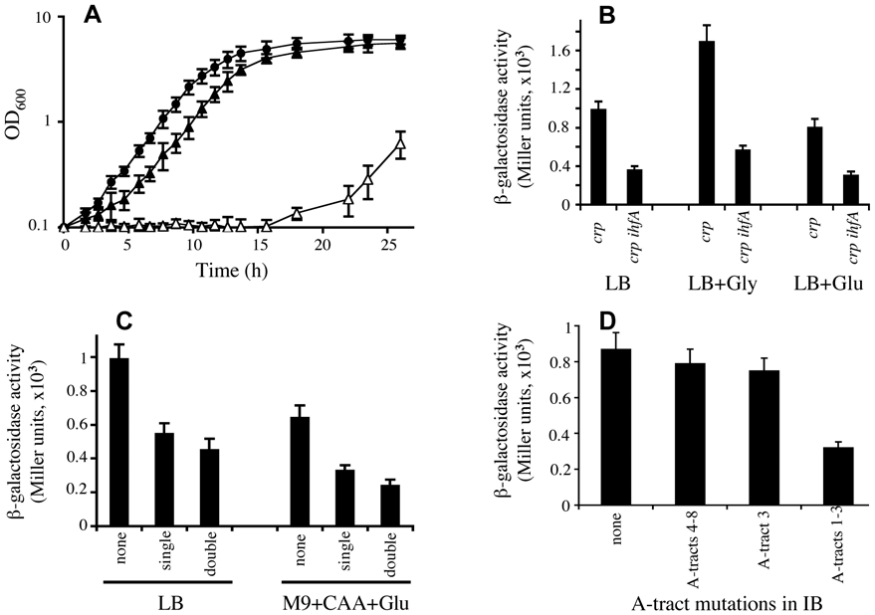
Dependency of *glpFK* promoter activation on IHF binding to IB (A–C) as well as the A-tract-promoting permanent bend in IB (D). (A) Growth of *crp* Glp^+^ (•), *ihfA* (σ) and *crp* Glp^+^ *ihfA* (▒) cells in liquid glycerol (1%) M9 minimal medium. (B) Effect of a host *ihfA* null mutation on IB:P*glpFK* activity. (C) Effect of IHF binding site mutations in IB on IB:P*glpFK* activity. ‘None’, ‘single’, and ‘double’ refer to no mutation, mutation of TCAA (−221 to −218 relative to +1 of P*glpFK*) to GTCT, and mutation of TCAA to GTCT as well as TT (−213 to −212) to GC in the IHF binding site located in IB, respectively (see Figure 1B). (D) *lacZ* expression measured by β-galactosidase assay for *crp* cells carrying an IB:P*glpFK*-*lacZ* fusion with various A-tract mutations in IB. The strain bearing altered A-tracts 4–8 includes the mutations shown in A-tracts 4–8. ‘none’, ‘A-tracts 4–8’, ‘A-tract 3’ and ‘A-tracts 1–3’ refer to no mutation, mutations in A-tracts 4 to 8, mutation in A-tract 3 and mutations in A-tracts 1 to 3, respectively (see Figure 1B).

As demonstrated above, the IB segment has the same ability as the intact IS5 to activate P*glpFK* in the *crp* genetic background. A putative IHF binding site is present in the middle of IB [25]. To determine if the host IHF protein plays a role in activation of P*glpFK*, we generated a null *ihfA* mutant in *wt* and *crp* Glp^+^ backgrounds. These mutant strains were compared for growth in M9 minimal medium with glycerol as the sole carbon source (Figure 5A). No obvious difference in growth was found between *wt* and *ihfA* mutant cells. However, growth of *crp* Glp^+^ cells was substantially reduced by the *ihfA* mutation.

To determine the effect of the loss of IHF on transcription of IB:P*glpFK*, an IB:P*glpFK*-*lacZ* fusion was transferred into a *crp ihfA* double mutant genetic background by P1 transduction. β-Galactosidase assays were performed after growing cells in LB media with or without glycerol or glucose. As shown in Figure 5B, the activity of IB:P*glpFK* was reduced ∼60% by the loss of IHF. Similar results were obtained when M9 + casamino acids + glucose medium was used (data not shown). These results are consistent with the growth data described above and indicate that IHF is required for maximal activation of P*glpFK* by the IB segment.

To determine if the putative IHF binding site in IB is responsible for IHF-mediated activation, we mutated the site as follows (see Figure 1A): (1) TCAA (−218 to −221, relative to +1 of P*glpFK*) to GTCT (single mutation) and (2) TCAA to GTCT together with TT (−212 to −213) to GC (double mutations). The activities of the IB:P*glpFK* constructs with any one of these mutated IHF binding sites were determined using transcriptional *lacZ* fusions (Figure 5C). When cells were grown in minimal or LB medium, these alterations resulted in 50–60% reductions in the activity of IB:P*glpFK* compared to the parental construct. These results clearly indicate that the IHF binding site in the middle of IB is required for activation of P*glpFK* by the host IHF protein.

To determine if an IHF binding site alone is capable of stimulating the activity of the *glpFK* promoter, an IHF binding site was introduced upstream of P*glpFK* by changing gccttgcagatta (−222 to −210 relative to +1 of P*glpFK*) to aatcaagcagtta (Figure S3A). The newly created site was at the same distance as that of the IHF site in IB:P*glpFK* relative to the promoter (Figure 1B and Figure S3A). Using a chromosomal *lacZ* fusion, the activity of P*glpFK* containing this upstream IHF binding site was examined in *wt* and *crp* cells grown in LB medium. The promoter activity was increased by ∼40% in *wt* cells but was not changed in *crp* cells (Figure S3B). These results show that (1) IHF can bind to the created binding site; (2) the binding of IHF is capable of partially stimulating P*glpFK* activity but only in the presence of Crp; and (3) IHF stimulation of IB:P*glpFK* in the absence of Crp is dependent not only on the IHF binding site present in IB but also on the adjacent sequences (e.g., the A-tracts) surrounding the site.

### Dependency of *glpFK* Operon Activation on the Permanent Bend in IS5

A characteristic of a permanent DNA bend is the presence of in-phase A-tracts of 3–6 tandem As. The permanent bend in the IB region of IS5 has been shown to be one of the largest bend angles in the *E. coli* chromosome [25].

To examine the dependency of *glpFK* expression in a *crp* Glp^+^ genetic background on the A-tracts in the IB region of IS5, three different sets of mutated A-tracts were constructed by changing specific As in these tracts to Cs or Gs (see Figure 1A). The mutated strains were then examined for expression using a P*glpFK*-*lacZ* reporter gene fusion. All three sets of A-tract mutations lowered the expression level. However, mutating the five upstream A-tracts (A-tracts 4–8, see Figure 1A) had a minimal effect on expression (Figure 5D). Mutation of A-tract 3 only (A-tract 3) also had a minimal effect. However, mutational alteration of three consecutive A-tracts in the downstream region (A-tracts 1–3) had a dramatic effect on gene expression. From these observations and those reported in the previous section, we conclude that (i) the downstream permanent bend is important for gene activation, (ii) the upstream A-tract region is of minimal importance for gene expression, and (iii) IHF binding and the three downstream A-tracts are roughly of equal importance. We can therefore account for IS5 activation of *glpFK* operon expression by IHF binding and the downstream A-tracts which apparently activate gene expression in an additive fashion, probably by bending the DNA.

To confirm the importance of both the A-tracts and the IHF binding site in IB to IS5 activation of P*glpFK*, the IB:P*glpFK*-*lacZ* fusion containing mutations in A-tracts 1–3 in IB was transferred into the *ihfA* genetic background. As shown in Figure S4, promoter activity was completely abolished. This experiment therefore shows that the activating effects of IHF and the downstream permanent bend are responsible for promoter activation, and that these two effects are additive.

As shown above, when the upstream 47 bps in the 177 bp fragment (IB) were deleted, 60% of the activation was retained. When the upstream A-tracts were disrupted by point mutations, 85% of the activation was retained (Figure 5D). There may therefore be a mild dependency of activation on these upstream sequences, but we do not know exactly why this difference was observed. Possibly, deletion of this upstream region has an indirect contextual effect on the downstream region that plays a dominant role in *glpFK* promoter activation.

### Effects of IS5 and IB on the Activity of the *lacZYA* Promoter (P*lac*)

To determine if IB can activate a different Crp-dependent promoter in a *crp* genetic background, IB was placed at positions −126.5 (the same relative position as in IB:P*glpFK*) and −178.5 (relative to +1 of P*lac*) upstream of P*lac*, yielding IB:P*lac* and IB:P*lac*', respectively. These two sites differ by 22 bps, are therefore should be in phase. The difference in activation of P*lac* would presumably reflect the distance from the activated promoter.

**Figure 6:**
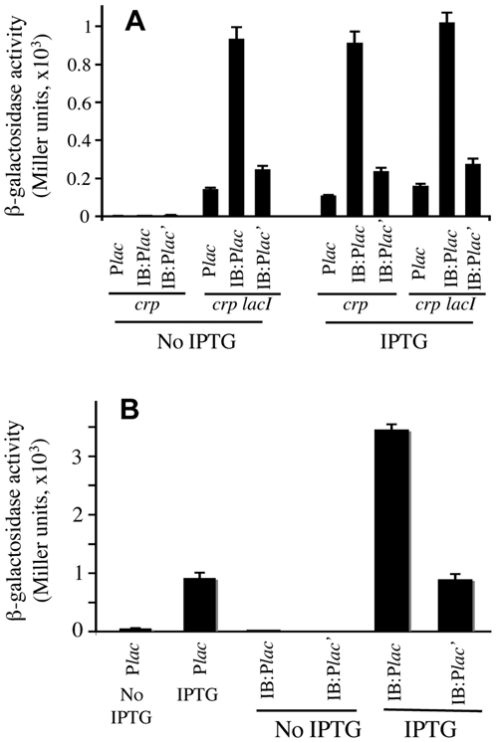
Effects of IB and its location on the activity of the *lacZYA* promoter (P*lac*). (A) Effect on P*lac* in the absence of Crp. (B) Effect on P*lac* in the presence of Crp. *crp*, *crp lacI*, and *wt* cells were grown in M9 minimal medium + 0.66% CAA + 1% glucose without or with IPTG (200 µM). In IB:P*lac* and IB:P*lac*', IB is located at −126.5 (the same relative position as in IB:P*glpFK*) and −178.5 upstream of the *lacZ* transcriptional start site, respectively.

Using chromosomal *lacZ* fusions, the activities of these constructs were examined in both *crp* and *crp lacI* cells grown in M9 medium + 0.66% casamino acids +1% glucose (Figure 6A). In the absence of IPTG, no appreciable activity was observed for P*lac* with or without the upstream IB sequence in *crp* cells (columns 1–3 of Figure 6A). However, in the presence of IPTG, IB increased the P*lac* activity 8 fold when it was located at −126.5, and 2 fold when it was located at −178.5 compared to P*lac* alone (columns 7–9 of Figure 6A). These two positions are in phase assuming 10.5 bp per turn in DNA. We also measured the activities of IB:P*lac* and IB:P*lac*' in *crp lacI* double mutant cells. As expected, regardless of the presence of IPTG, they behaved similarly as in *crp* cells with IPTG (i.e., 8 and 2 fold increased activation for IB:P*lac* and IB:P*lac*', respectively). The entire IS5 element showed the same ability as the IB element to activate P*lac* (data not shown). These results indicate that (i) IS5 or IB can at least partially replace the function of Crp in activating P*lac*; (ii) a proper location of these fragments is important for activation of gene expression in the absence of Crp; and (iii) in contrast to the *glpFK* system, activation is fully blocked by operator-bound LacI.

We further measured IB effects on P*lac* activity in wild type (*crp* ^+^) cells grown in the same M9 minimal medium as above. In the absence of IPTG, the activities of P*lac* with or without the upstream IB were extremely low (20 Miller units or less) (Columns 1, 3 and 4 of Figure 6B). In the presence of IPTG, IB increased the *lac* promoter activity ∼2.5 fold when it was located at −126.5 compared to the *lac* promoter alone. No increased P*lac* activity was observed when IB was located at −178.5 upstream of the promoter. These results indicate that (i) LacI still blocks the activation of P*lac* by both Crp and IB, and (ii) when present at an appropriate position, IB is still capable of enhancing the activity of P*lac* in the presence of Crp. In other words, IB and Crp activate P*lac* in an additive fashion. This is different from IB activation of P*glpFK*, in which case, the presence of Crp did not further elevate the activity of IB:P*glpFK* (see Figure 4B).

## Discussion

We have demonstrated that a transposon, IS5, is capable of activation of the *glpFK* operon, rendering *E. coli* cells capable of utilizing glycerol in the absence of Crp. Transposable elements have been found to activate transcription of adjacent genes by introducing complete or partial promoters located within the element or by disrupting or displacing a negative element that normally blocks transcription [3],[4]. The mechanism of *glpFK* activation by IS5 is distinct from these reported mechanisms. In this case, a part of IS5, which is proximal to the adjacent gene and harbors unique sequences (A-tracts and an IHF binding site), can functionally replace Crp and directly activate the native downstream promoter.

Prior to the work reported here, the best-characterized example of transposon-mediated gene activation was of the β-glucoside (*bgl*) operon in *E. coli* (see Introduction) [8]–[12]. This system shows the following characteristics: 1) The *bgl* operon is not active in wild-type *E. coli*. 2) Either IS1 or IS5 can activate *bgl* operon expression. 3) When it is activated, *bgl* expression is subject to catabolite control by Crp as well as inducer sensitivity by an anti-termination mechanism. 4) Activation by the IS element occurs by an enhancer-type mechanism. 5) The IS element can activate when either upstream or downstream of the promoter, and in either orientation. 6) Activation is at least partially dependent on the IS-encoded transposase. 7) Activation is dependent on relief of repression by H-NS. 8) Although the most frequent mechanism for activation involves insertion sequence elements, activation can also be caused by mutations in *gyrA* or *gyrB* [26], *hns* [27], *bglJ* [28],[29], *leuO* [30] or the Crp binding site of the *bgl* promoter [31],[32].

In all of these respects, activation of *glpFK* appears to differ from that of the *bgl* operon: 1) The *glpFK* operon is active in wild-type *E. coli*. 2) Only IS5 has been observed to activate *glpFK* operon expression in a *crp* genetic background. 3) Once activated, the operon is expressed independently of Crp and largely independently of the *glp* regulon repressor, GlpR. 4) There is no evidence that IS5 acts on P*glpFK* by an enhancer-type mechanism. 5) Only a single site of insertion and only one orientation were demonstrated, and this site proved to be the IS5 tetranucleotide target sequence, CTAA, located at a specific position upstream of the promoter. 6) Activation is independent of the IS5-encoded transposase. 7) IHF, but not H-NS, is required for maximal gene activation. 8) Except for IS5 insertion, no other mutations have been identified to account for activation of *glpFK* expression.

We identified a short sequence in IS5 (IB) that is fully responsible for P*glpFK* activation. This sequence includes a permanent bend and an IHF binding site. An IHF binding site has been found to be present in the ends of the IS1 element, but several lines of evidence have shown that this IHF site does not play a role in gene activation although it bends the DNA [33]. In the present study, we showed that the IHF binding site in IS5 plays an important role since an *ihfA* null mutation or alteration of the IHF binding site partially abolished activation to the same degree. No function for the IB region had been recognized prior to our studies. It may have evolved specifically for the purpose of gene activation, for another unrecognized purpose, or for both.

We further showed that DNA phasing is important since the insertion of 5 bps (but not 10 bps) between the activating element and P*glpFK* abolished activation. Preliminary evidence suggested that the C-terminal domain of the α-subunit of RNA polymerase is not required for activation (unpublished results), but a DNA looping mechanism is nevertheless proposed.

The activating IB region contains as many as 10 A-tracts, which in general can induce permanent bends in DNA (see Figure 5D) [25],[34] and can increase promoter activity [35],[36]. In one case, transcriptional activation involves binding of the DNA to the C-terminal domain of the RNA polymerase α-subunit (35). In view of our results, it seems likely that the permanent bend in the downstream region of IS5 together with bound IHF activates *glpFK* promoter activity at least in part by bending the upstream DNA. A direct interaction of IHF with the transcriptional initiation complex is possible [37].

Although this is the first study to show that unique sequences inside a transposon are necessary and sufficient to activate a downstream silent promoter, similar mechanisms of gene activation may occur for other operons (see Figure 6 and unpublished results). These include the *lacZYA*, *fucAO* and *flhDC* operons [13],[14 and observations reported here]. On the other hand, the isolation and analysis of mutations that allow *E. coli crp* mutants to grow on several other Crp-dependent carbon sources [38] indicate that IS-mediated gene activation is not the only mechanism available to *E. coli*. We propose that IS5 insertion under the control of a host regulatory protein represents just one of many mechanisms of operon adaptive activation that will prove to occur under stressful conditions.

## Materials and Methods

### Bacterial Strains and Growth Conditions

Strains and oligonucleotides used in this study are described in Table S1 and Table S2, respectively. The *crp*, *glpR*, *crp* Glp^+^ and *crp glpR* Glp^+^ mutants, derived from *E. coli* K-12 strain BW25113, were constructed previously [21]. The *crp lacI* double mutant was made by transferring the *lacI* insertion mutation from MG1655 [39] to a BW25115 *crp* deletion background using P1 transduction. The *hns* and *ihfA* isogenic deletion mutant was derived from the same *wt* strain BW25113 using the method of Datsenko and Wanner [40]. Double and triple mutants, *crp hns*, *crp* Glp^+^ *hns*, *crp ihfA* and *crp* Glp^+^ *ihfA*, were made by P1 transduction. The detailed procedures for generating single or double mutants are described in the reference 21. Strain BW25113 is deleted for the *lacZ* gene and the *araBAD* operon encoding proteins required for L-arabinose metabolism [40]. All mutants were verified by PCR. The strains were cultured in LB or minimal M9 media with various carbon sources at 37°C or 30°C. When appropriate, kanamycin (Km; 25 µg/ml), or ampicillin (Ap; 100 µg/ml) was added to the media.

### Glycerol Kinase Activity Assay

The activity of glycerol kinase encoded by *glpK* was determined using [1,3-^14^C]glycerol as substrate. Cells were cultured in LB with or without 1% glycerol, and cellular extracts were prepared using a French press and the subsequent ultracentrifugation. Glycerol phosphorylation by GlpK in the extracts was quantitated as described previously [41].

### Site-Directed Mutagenesis

Using the quick-change site-directed mutagenesis kit (Stratagene), the following modifications were made in IS5, its IB region or the *glpFK* promoter region: (i) removal of the *EcoR*I site in IS5 by changing A (135 bp from the 5′ end) to G; (ii) insertion of a 5-bp (tacct) or a 10-bp (taccttacct) oligonucleotide at −117.5 (relative to +1 of P*glpFK*) in the IB:P*glpFK* junctional region (see Figure 1B); (iii) mutation of the IHF binding site in IB by changing TCAA to GTCT (−221 to −218, relative to +1 of P*glpFK*) and TT (−213 to −212, relative to +1 of P*glpFK*) to GC (see Figure 1B); iv) mutations of individual or multiple A-tracts in IB by changing one or two As to Cs or Gs (see Figure 1B). All the mutation primers are listed in Table S2. Sequence alterations were confirmed by DNA sequencing.

### Real Time (RT) PCR

RT-PCR was performed as described previously [42]. Cells were grown in LB with or without 1% glycerol. Total RNA was prepared using an EZgene™ RNA purification kit (Biomiga). The contaminated chromosomal DNA in RNA samples was removed by DNase I treatment. cDNAs were synthesized using an Invitrogen superscript first-strand synthesis kit. *polA*, encoding DNA polymerase I, was included as an internal control [43]. Primers used for RT-PCR are listed in Table S2. RT-PCR was carried out with a LightCycler instrument (Roche).

### Chromosomal *lacZ* Fusions and β-Galactosidase Assays

The *glpFK* promoter region (−203 to +67 relative to the transcriptional start site), with or without the upstream IS5 element or part of the IS5 element, was PCR amplified from the chromosome of *crp* or *crp* Glp^+^ cells. The PCR products were digested with *BamH*I and *EcoR*I, gel purified using an EZgene™ gel purification kit (Biomiga), and then ligated into the same sites of pRS551 [44]. Transcriptional *lacZ* fusions present in the pRS551 plasmids were integrated into the lambda attachment site (*attB*) in the *E. coli* chromosome using the method of Simons et al. [44]. P1 transduction was used to transfer a chromosomal promoter:*lacZ* fusion from one strain to another strain.

To add an IHF binding site upstream of the *glpFK* promoter at the same relative distance as in IB:P*glpFK*, a separate P*glpFK* (−220 to +67) was amplified from the *wt* chromosome using primers PglpFK_IHF-F and PglpFK-R1 (Table S2). An IHF binding site (aatcaagcagtta) was present at the 5′ end of primer PglpFK_IHF-F. The PCR products that contained the IHF binding site were fused to a promoter-less *lacZ* gene, and the resultant transcriptional promoter:*lacZ* fusion was moved to the chromosome as described above (see Figure S3).

To make chromosomal P*lac*-*lacZ*, P*lacZYA* (−126 to +38 or −178 to +38) was first cloned into the *BamH*I and *EcoR*I sites of pRS551. The entire IS5 or IB was inserted into the *BamH*I site of the plasmid so that IS5 or IB was located at −126.5 or −178.5, relative to +1 of P*lacZYA*. The fusions of P*lac* with or without IS5 or IB to *lacZ* were integrated to the chromosome of *crp* cells. Primers used for RT-PCR and *lacZ* fusion construction are listed in Table S2.

β-Galactosidase assays, conducted after growth in either LB or M9 minimal media ± 1% glycerol, 1% glucose or 0.66% casamino acids (CAA), were as described by Miller [45]. CAA was added to improve growth of *crp* cells in minimal media.

### RNA Ligase Mediated PCR

RNA ligase mediated PCR was used to determine the transcriptional start site of the *glpFK* operon as described by Bensing et al. [46]. Cells were grown in LB with or without 1% glycerol. The total RNA was prepared using a Biomiga RNA purification kit and treated with or without tobacco acid pyrophosphatase (TAP). The RNA oligonucleotide adaptor (Table S2) was ligated to all RNAs, and the 5′-end of the *glpFK* cDNA was synthesized using an oligonucleotide (PglpFK-extn-R) complementary to nucleotides 118–139 (Table S2) downstream of the ATG start codon. cDNA was amplified using primers PglpFK-extn-F and PglpFK-extn-R, and the PCR product was sequenced using the same primer (PglpFK-extn-R) as for cDNA synthesis. The transcriptional start site, i.e., the junction between the cDNA and the RNA oligonucleotide, was determined by sequencing.

## Supporting Information

Figure S1 Real-time PCR analysis of effects of the *hns* mutation on *glpFK* expression in *wt*, *crp* and *crp* Glp^+^ backgrounds. Cells were grown in LB liquid medium.
(0.10 MB TIF)

Figure S2 Effects of IS5 and various regions within IS5 on expression of the downstream *glpFK* promoter in *crp* cells lacking IS5. ‘IS’, ‘Pless’, ‘IB’, ‘none’, and ‘178’, refer to transcriptional *lacZ* fusions for IS5:PglpFK, promoter-less IS5:P*glpFK*, IB:P*glpFK*, native P*glpFK*, and 178bp:P*glpFK*, respectively (see Figure 1A). *E. coli* strain B has been reported to lack IS5 in its genome [24]. The *crp* mutation was transferred to strain B by P1 transduction. The promoter:*lacZ* fusions described above were individually transferred to strain B *crp* cells from BW25113 by P1 transduction. For β-galactosidase assays, *E. coli* strain B *crp* cells containing these *lacZ* fusion constructs were grown in LB with shaking.
(0.12 MB TIF)

Figure S3 Effect of addition of an IHF binding site upstream of P*glpFK* on promoter activity. (A) The *glpFK* promoter region showing that an IHF binding site is added upstream of P*glpFK* by changing gccttgcagatta (−222 to −210) to aatcaagcagtta. The newly added IHF binding site is located at the same relative distance as in IB:P*glpFK*. (B) Effect of the added IHF site on P*glpFK* activity in *wt* and *crp* cells grown in LB medium. P*glpFK* and P*glpFK*_IHF refer to the transcriptional *lacZ* fusions for native P*glpFK* and the same promoter with an upstream IHF binding site, respectively.
(0.37 MB TIF)

Figure S4 Effect of A-tract mutations in IB on *glpFK* promoter activity in the *ihfA* genetic background. The IB:P*glpFK-lacZ* fusion with or without mutations in A-tracts 1–3 in IB was transferred into the *crp ihfA* double genetic background. The cells were cultured in LB medium.
(0.12 MB TIF)

Table S1 Strains used in this study.
(0.06 MB DOC)

Table S2 Oligonucleotides used in this study.
(0.08 MB DOC)
