# Supplementary figures and images for: A Novel Mechanism of Transposon-Mediated Gene Activation
Source: PLoS Genet. 2009 Oct 16;5(10):e1000689. doi: 10.1371/journal.pgen.1000689 (PMC2753651; doi:10.1371/journal.pgen.1000689)

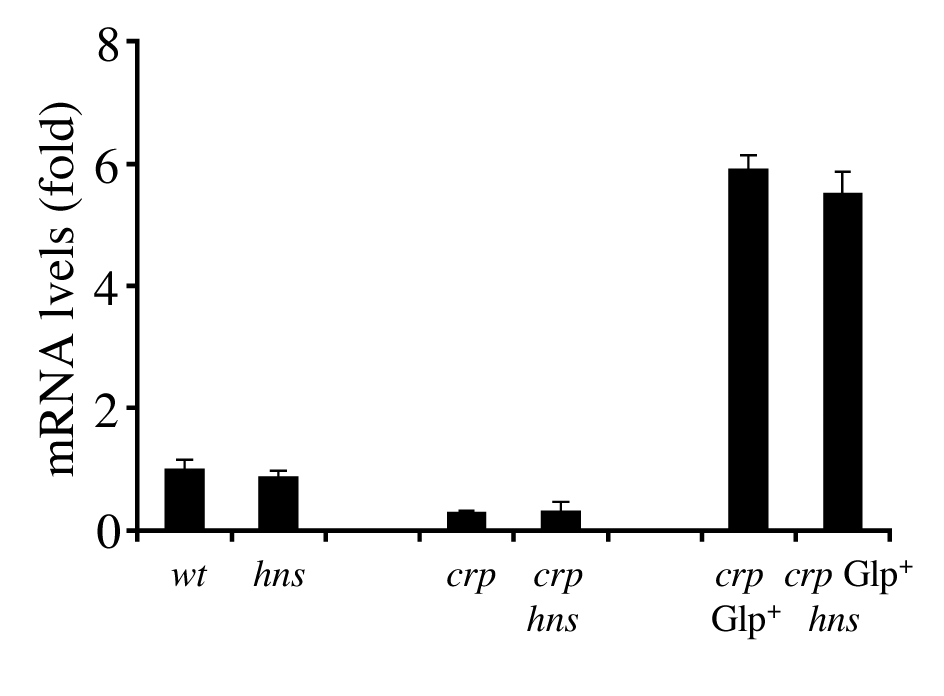

Supplement: Figure S1 — Real-time PCR analysis of effects of the hns mutation on glpFK expression in wt, crp and crp Glp+ backgrounds. Cells were grown in LB liquid medium. (0.10 MB TIF) [file pgen.1000689.s001.tif]

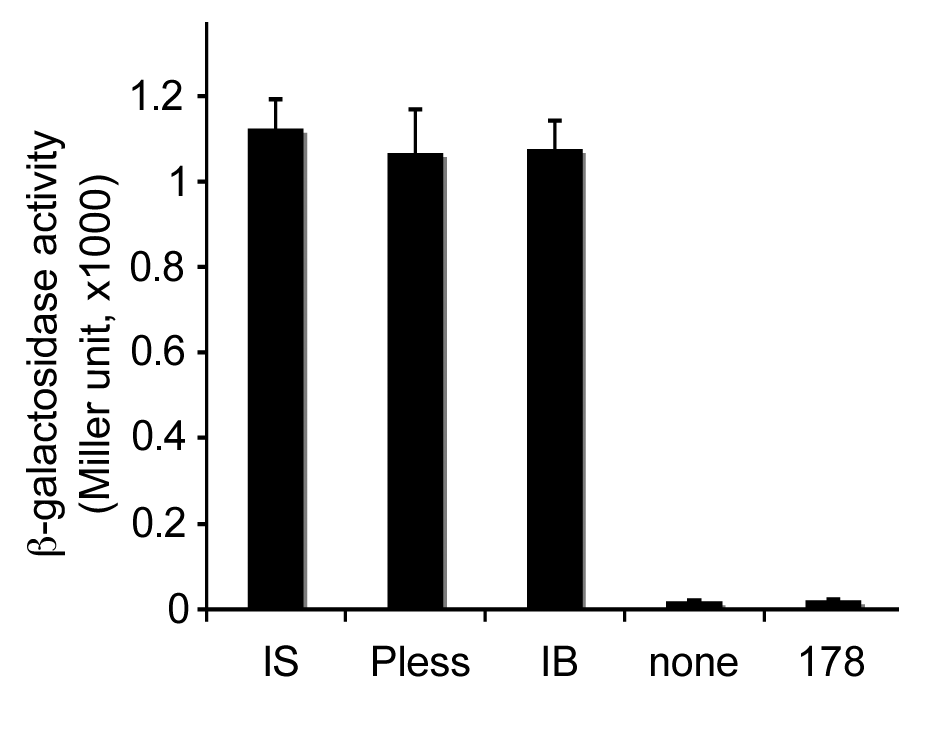

Supplement: Figure S2 — Effects of IS5 and various regions within IS5 on expression of the downstream glpFK promoter in crp cells lacking IS5. ‘IS’, ‘Pless’, ‘IB’, ‘none’, and ‘178’, refer to transcriptional lacZ fusions for IS5:PglpFK, promoter-less IS5:PglpFK, IB:PglpFK, native PglpFK, and 178bp:PglpFK, respectively (see Figure 1A). E. coli strain B has been reported to lack IS5 in its genome [24]. The crp mutation was transferred to strain B by P1 transduction. The promoter:lacZ fusions described above were individually transferred to strain B crp cells from BW25113 by P1 transduction. For β-galactosidase assays, E. coli strain B crp cells containing these lacZ fusion constructs were grown in LB with shaking. (0.12 MB TIF) [file pgen.1000689.s002.tif]

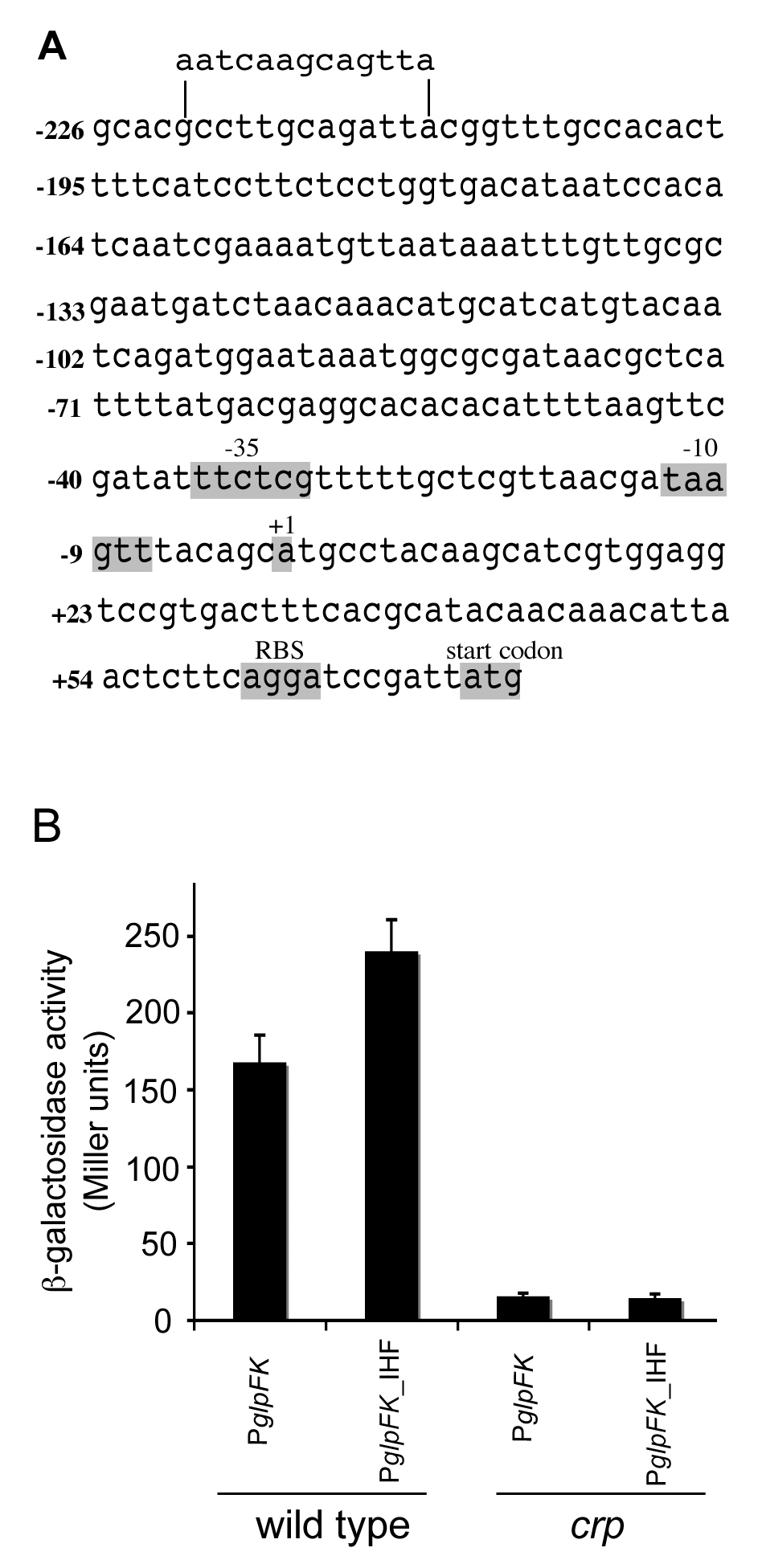

Supplement: Figure S3 — Effect of addition of an IHF binding site upstream of PglpFK on promoter activity. (A) The glpFK promoter region showing that an IHF binding site is added upstream of PglpFK by changing gccttgcagatta (−222 to −210) to aatcaagcagtta. The newly added IHF binding site is located at the same relative distance as in IB:PglpFK. (B) Effect of the added IHF site on PglpFK activity in wt and crp cells grown in LB medium. PglpFK and PglpFK_IHF refer to the transcriptional lacZ fusions for native PglpFK and the same promoter with an upstream IHF binding site, respectively. (0.37 MB TIF) [file pgen.1000689.s003.tif]

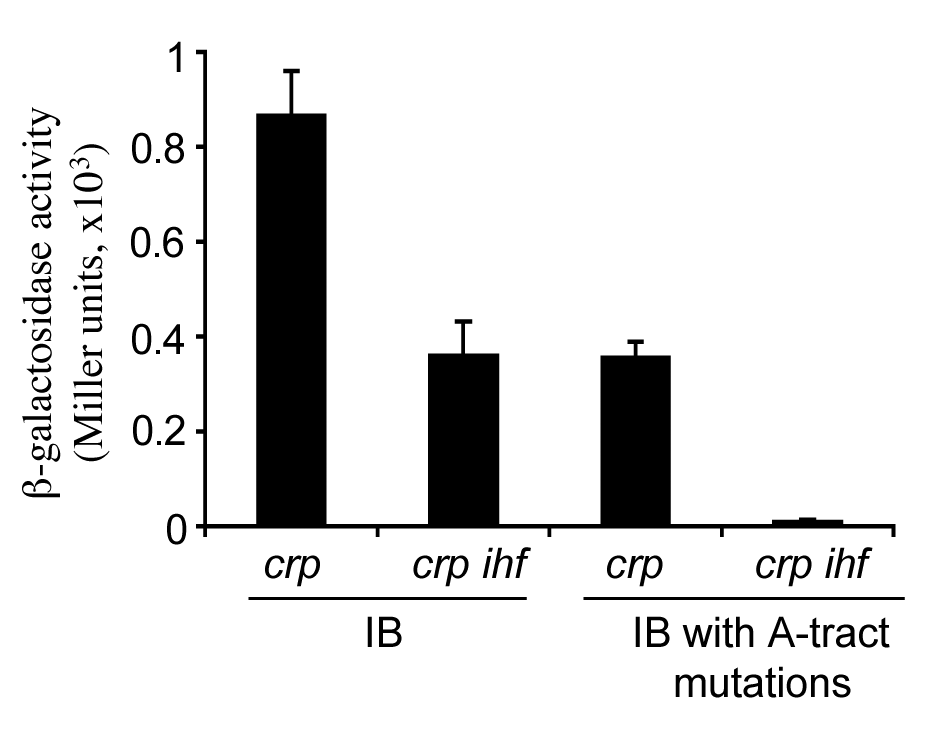

Supplement: Figure S4 — Effect of A-tract mutations in IB on glpFK promoter activity in the ihfA genetic background. The IB:PglpFK-lacZ fusion with or without mutations in A-tracts 1–3 in IB was transferred into the crp ihfA double genetic background. The cells were cultured in LB medium. (0.12 MB TIF) [file pgen.1000689.s004.tif]
