# Supplementary material for: A Novel Mechanism of Transposon-Mediated Gene Activation
Source: PLoS Genet. 2009 Oct 16;5(10):e1000689. doi: 10.1371/journal.pgen.1000689 (PMC2753651; doi:10.1371/journal.pgen.1000689)
Supplement: Table S1 — Strains used in this study. (0.06 MB DOC) [file pgen.1000689.s005.doc]

# Table S1. Strains used in this study

| **Strains/Plasmids** | **Genotype or description** | **Reference or source** |
| --- | --- | --- |
| **Strains** |  |  |
| BW25113 | *lacI*^q^ *rrnB*_T14_ Δ*lacZ*_WJ_ Δ*hsdR*514 Δ*araBAD*_AH33_ Δ*rhaBAD*_LD78_ | Ref. 31 |
| *crp* | Δ*crp* in BW25113 | Ref. 29 |
| *glpR* | Δ*glpR* in BW25113 | Ref. 21 |
| *crp* Glp^+^ | Δ*crp* carrying IS5 upstream of P*glpFK* | Ref. 21 |
| *crp glpR* | Δ*crp* Δ*glpR* in BW25113 | Ref. 21 |
| *crp glpR* Glp^+^ | Δ*glpR* in *crp* Glp^+^ | Ref. 21 |
| *lacI* | Δ*lacI* in MG1655 | Ref. 30 |
| *crp lacI* | Δ*crp* Δ*lacI* in BW25113 | This study |
| *hns* | Δ*hns* in BW25113 | This study |
| *crp hns* | Δ*crp* Δ*hns* in BW25113 | This study |
| *crp* Glp^+^ *hns* | Δ*hns* in *crp* Glp^+^ | This study |
| *ihfA* | Δ*ihfA* in BW25113 | This study |
| *crp ihfA* | Δ*crp* Δ*ihfA* in BW25113 | This study |
| *crp* Glp^+^ *ihfA* | Δ*ihfA* in *crp* Glp^+^ | This study |
| *wt*(P*glpFK*-*lacZ*) | BW25115 containing P*glpFK*-*lacZ* fusion at the *attB* site | This study |
| *crp*(P*glpFK*-*lacZ*) | Δ*crp* containing P*glpFK*-*lacZ* fusion at the *attB* site | This study |
| *glpR*(P*glpFK*-*lacZ*) | Δ*glpR* containing P*glpFK*-*lacZ* fusion at the *attB* site | This study |
| *crp glpR*(P*glpFK*-*lacZ*) | Δ*crp* Δ*glpR* containing P*glpFK*-*lacZ* fusion at the *attB* site | This study |
| *wt*(IS5:P*glpFK*-*lacZ*) | BW25115 containing IS5:P*glpFK*-*lacZ* fusion at the *attB* site | This study |
| *crp*(IS5:P*glpFK*-*lacZ*) | Δ*crp* containing IS5:P*glpFK*-*lacZ* fusion at the *attB* site | This study |
| *glpR*(IS5:P*glpFK*-*lacZ*) | Δ*glpR* containing IS5:P*glpFK*-*lacZ* fusion at the *attB* site | This study |
| *crp glpR*(IS5:P*glpFK*-*lacZ*) | Δ*crp* Δ*glpR* containing promoter-less IS5:P*glpFK*-*lacZ* fusion at the *attB* site | This study |
| *wt*(Pless-IS5:P*glpFK*-*lacZ*) | BW25115 containing promoter-less IS5:P*glpFK*-*lacZ* fusion at the *attB* site | This study |
| *crp*(Pless-IS5:P*glpFK*-*lacZ*) | Δ*crp* containing promoter-less IS5:P*glpFK*-*lacZ* fusion at the *attB* site | This study |
| *glpR*(Pless-IS5:P*glpFK*-*lacZ*) | Δ*glpR* containing promoter-less IS5:P*glpFK*-*lacZ* fusion at the *attB* site | This study |
| *crp glpR*(Pless-IS5:P*glpFK*-*lacZ*) | Δ*crp* Δ*glpR* containing promoter-less IS5:P*glpFK*-*lacZ* fusion at the *attB* site | This study |
| *wt*(IB:P*glpFK*-*lacZ*) | BW25115 containing the IB:P*glpFK*-*lacZ* fusion at the *attB* site | This study |
| *crp*(IB:P*glpFK*-*lacZ*) | Δ*crp* containing the IB:P*glpFK*-*lacZ* fusion at the *attB* site | This study |
| *glpR*(IB:P*glpFK*-*lacZ*) | Δ*glpR* containing the IB:P*glpFK*-*lacZ* fusion at the *attB* site | This study |
| *crp glpR*(IB:P*glpFK*-*lacZ*) | Δ*crp* Δ*glpR* containing IB:P*glpFK*-*lacZ* fusion at the *attB* site | This study |
| *crp*(IB:P*lac*-*lacZ*) | Δ*crp* containing the IB:P*lac*-*lacZ* with IB at -126.5 | This study |
| *crp*(IB:P*lac’*-*lacZ*) | Δ*crp* containing the IB:P*lac*-*lacZ* with IB at -178.5 | This study |
| *crp*(IS5:P*lac*-*lacZ*) | Δ*crp* containing the IS5:P*lac*-*lacZ* with IB at -126.5 | This study |
| *crp*(IS5:P*lac’*-*lacZ*) | Δ*crp* containing the IS5:P*lac*-*lacZ* with IB at -178.5 | This study |
|  |  |  |
| **Plasmids** |  |  |
| pRS551-P*glpFK* | pRS551 containing P*glpFK*-*lacZ* fusion | This study |
| pRS551-IS5:P*glpFK* | pRS551 containing IS5:P*glpFK*-*lacZ* fusion | This study |
| pRS551Pless-IS5:P*glpFK* | pRS551 containing Promoter-less IS5:P*glpFK*-*lacZ* fusion | This study |
| pRS551-IB:P*glpFK* | pRS551 containing IB:P*glpFK*-*lacZ* fusion | This study |
| pRS551-P*lacZYA* | pRS551 containing P*lacZYA*-*lacZ* fusion | This study |
| pRS551-IS5:P*lac* | pRS551 containing IS5:P*lac*-*lacZ* fusion with IS5 at -126.5 | This study |
| pRS551-IS5:P*lac’* | pRS551 containing IS5:P*lac*-*lacZ* fusion with IS5 at -178.5 | This study |
| pRS551-IB:P*lac* | pRS551 containing IB:P*lac*-*lacZ* fusion with IS5 at -126.5 | This study |
| pRS551-IB:P*lac’* | pRS551 containing IB:P*lac*-*lacZ* fusion with IS5 at -178.5 | This study |
