# Supplementary material for: A Novel Mechanism of Transposon-Mediated Gene Activation
Source: PLoS Genet. 2009 Oct 16;5(10):e1000689. doi: 10.1371/journal.pgen.1000689 (PMC2753651; doi:10.1371/journal.pgen.1000689)
Supplement: Table S2 — Oligonucleotides used in this study. (0.08 MB DOC) [file pgen.1000689.s006.doc]

# Table S2. Oligonucleotides used in this study

| **Name** | **Sequence** | **Use** |
| --- | --- | --- |
| ihfA1-P1 | agagcgattccaggcatcattgagggattgaacctatggcgcttagtgtaggctggagctgcttcg | *ihfA* mutation |
| ihfA2-P2 | ggccgcagagcggcctttttagttagatcagattactcgtctttgcatatgaatatcctccttag | *ifhA* mutation |
| HNS1-P1 | ctcaacaaaccaccccaatataagtttgagattactacaatgagcgtgtaggctggagctgcttc | *hns* mutation |
| HNS2-P2 | gcaagtgcaatctacaaaagattattgcttgatcaggaaatcgtcatatgaatatcctccttag | *hns* mutation |
| IS5_EcoR-F | taccttcgccgacagtgagttcagcagtaagcgccgt | Deletion of *EcoR*I site in IS5 |
| IS5_EcoR-R | acggcgcttactgctgaactcactgtcggcgaaggta | Deletion of *EcoR*I site in IS5 |
| PglpFK-F1 | aaagaattcgccacacttttcatccttctcc | P*glpFK* cloning |
| PglpFK-R1 | atcggatcctgaagagttaatg | P*glpFK* cloning |
| IS5-ER-F1 | aaagaattcaatgatacgtcagtgggagag | IB:PglpFK cloning |
| Plac-Eco-126 | attgaattcagctggcacgacaggtttcc | Cloning a small P*lac* to pRS551 |
| Plac-Eco-178 | aaagaattcgcgcccaatacgcaaacc | Cloning a big P*lac* to pRS551 |
| Plac-Bam | ataggatccagctgtttcctgtgtgaaattg | Cloning P*lac* to pRS551 |
| IB-Eco-F | atagaattccaatgatacgtcagtgggagag | Cloning IB into pRS551-P*lac* |
| IS5(IB)-Eco-R | aatgaattctgcgaataagcggggaaattc | Cloning IS5 or IB into pRS551-P*lac* |
| IS5-Eco-F | attgaattcgttgcgcgaatgatctaaggaag | Cloning IS5 into pRS551-P*lac* |
| RNA oligo | guauugcgguacccuuguacg | RNA ligase mediated RT-PCR |
| PglpFK-extn-F | gtattgcggtacccttgtacg | Amplification of 5’ end region of *glpFK* cDNA |
| PglpFK-extn-R | atgacactgatttcccactgacc | Amplification of 5’ end region of *glpFK* cDNA |
| IHF-mod1-F | gaaacggtctaaataggctgatgtctggcatttacgggagaaaaaatcg | Modification of TCAA to GTCT in IHF binding site of IB |
| IHF-mod1-R | cgattttttctcccgtaaatgccagacatcagcctatttagaccgtttc | Modification of TCAA to GTCT in IHF binding site of IB |
| IHF-mod2-F | cggtctaaataggctgattcaaggcagctacgggagaaaaaatcgg | Modification of TT to GC in IHF binding site of IB |
| IHF-mod2-R | ccgattttttctcccgtagctgccttgaatcagcctatttagaccg | Modification of TT to GC in IHF binding site of IB |
| IB-Atract-1F | caaggcatttacgggagaaccaatcggctcaaacatgaag | A-tract mutation in IB |
| IB-Atract-1R | cttcatgtttgagccgattggttctcccgtaaatgccttg | A-tract mutation in IB |
| IB-Atract-2F | aaaccatggatgactgagtcagccgagaag | A-tract mutation in IB |
| IB-Atract-2R | ataccatggtcttcatgtctgagccgattggttctc | A-tract mutation in IB |
| IB-Atract-3F | aaaggtacctggcgaagacacggtctacctaggctgattcaaggcatttacgg | A-tract mutation in IB |
| IB-Atract-3R | aatggtaccgcgtgatccccagttggtagtgagatctctcccactgacg | A-tract mutation in IB |
| IB-ins-5F | cgcaccttccctaacaaactacctatgcatcatgtacaatcag | 5 bp insertion between IB and P*glpFK* |
| IB-ins-5R | ctgattgtacatgatgcataggtagtttgttagggaaggtgcg | 5 bp insertion between IB and P*glpFK* |
| IB-ins-10F | cttccctaacaaactaccttacctatgcatcatgtacaatcag | 10 bp insertion between IB and P*glpFK* |
| IB-ins-10R | ctgattgtacatgatgcataggtaaggtagtttgttagggaag | 10 bp insertion between IB and P*glpFK* |
| PglpFK_IHF-F | aaagaattcaatcaagcagttacggtttgccacacttttcatcc | Addition of an IHF site upstream of P*glpFK* |
| RTglpA-F | cgactcgcaatcaagtgacg | RT-PCR for *glpA* |
| RTglpA-R | caatgcgtttcaggatctgg | RT-PCR for *glpA* |
| RTglpE-F | aaagaggcggtgctggtcg | RT-PCR for *glpE* |
| RTglpE-R | tagaccacatcgtagccctg | RT-PCR for *glpE* |
| RTglpF-F | tttcttcggtgtgggttgcg | RT-PCR for *glpFK* |
| RTglpF-R | cgaaacaggcaaacagccac | RT-PCR for *glpFK* |
| RTglpR-F | ctggtagagcatttctccgtc | RT-PCR for *glpR* |
| RTglpR-R | gatttgctccgccactttgc | RT-PCR for *glpR* |
| RTglpT-F | ccgacttatcgtcgattgcg | RT-PCR for *glpTQ* |
| RTglpT-R | ccgataccgaacccatgatg | RT-PCR for *glpTQ* |
